# Fuzzy-Assisted Mobile Edge Orchestrator and SARSA Learning for Flexible Offloading in Heterogeneous IoT Environment

**DOI:** 10.3390/s22134727

**Published:** 2022-06-23

**Authors:** Tran Trong Khanh, Tran Hoang Hai, Md. Delowar Hossain, Eui-Nam Huh

**Affiliations:** 1 Department of Computer Science and Engineering, Kyung Hee University, Yongin 17104, Korea; khanhtrannnn@khu.ac.kr (T.T.K.); delowar@khu.ac.kr (M.D.H.); 2 School of Information and Communication Technology, Hanoi University of Science and Technology, Hanoi 100000, Vietnam; haith@soict.hust.edu.vn

**Keywords:** Internet of Things, multi-access edge computing, task offloading, mobile edge orchestrator, fuzzy logic, SARSA

## Abstract

In the era of heterogeneous 5G networks, Internet of Things (IoT) devices have significantly altered our daily life by providing innovative applications and services. However, these devices process large amounts of data traffic and their application requires an extremely fast response time and a massive amount of computational resources, leading to a high failure rate for task offloading and considerable latency due to congestion. To improve the quality of services (QoS) and performance due to the dynamic flow of requests from devices, numerous task offloading strategies in the area of multi-access edge computing (MEC) have been proposed in previous studies. Nevertheless, the neighboring edge servers, where computational resources are in excess, have not been considered, leading to unbalanced loads among edge servers in the same network tier. Therefore, in this paper, we propose a collaboration algorithm between a fuzzy-logic-based mobile edge orchestrator (MEO) and state-action-reward-state-action (SARSA) reinforcement learning, which we call the Fu-SARSA algorithm. We aim to minimize the failure rate and service time of tasks and decide on the optimal resource allocation for offloading, such as a local edge server, cloud server, or the best neighboring edge server in the MEC network. Four typical application types, healthcare, AR, infotainment, and compute-intensive applications, were used for the simulation. The performance results demonstrate that our proposed Fu-SARSA framework outperformed other algorithms in terms of service time and the task failure rate, especially when the system was overloaded.

## 1. Introduction

In recent years, the development of heterogeneous fifth-generation (5G) networks has led to the rapid evolution of modern high technologies and has changed people’s daily lives by providing various high-demand and intensive applications (e.g., virtual/augmented reality (VR/AR), the internet of vehicles, mobile healthcare, cloud gaming, face/fingerprint recognition, industrial robotics, video streaming analysis, and autonomous driving) [1,2,3]. These applications generate a vast amount of data and require a fast response time and large resource capacities. Consequently, there is a significant burden on resource-constrained IoT devices to handle these heavy computational demands and to accomplish requests quickly. However, these devices are insubstantial with regard to their battery life, computing capabilities, and storage capacity, and are ineffectual when performing a high number of intensive tasks [4]. Therefore, it is crucial to offload the tasks to other powerful remote computing infrastructures.

In conventional cloud computing, mobile cloud computing (MCC) is a prominent model, supporting computational offloading for mobile devices [5]. By taking advantage of the enormous computing capabilities of MCC over a wide area network (WAN), user devices can send requests to powerful global cloud servers to utilize their rich computational resources for task processing. However, the long distance between the devices and core network in the MCC network not only causes high transmission delays, data losses, and significant energy consumption, it also limits the context-awareness of applications [6]. As a result, MCC is unable to meet the standard requirements of delay-sensitive and real-time applications in heterogeneous IoT environments.

To cope with these challenges, multi-access edge computing (MEC) [7], formerly mobile edge computing, is a European Telecommunications Standards Institute (ETSI)-proposed network architecture that brings the servers with cloud computing properties closer to the IoT devices and deploys them at the edge of the network [8]. Unlike the centralized nature of MCC, MEC deploys a dense and decentralized peer-to-peer network where the edge servers are allocated in a distributed manner. MEC provides ultra-low latency and a high-bandwidth environment which can be leveraged by IoT applications. Moreover, MEC enhances the quality of experience (QoE) and meets the requirements of the quality of services (QoS); for example, it provides a low execution time and reasonable energy consumption. Additionally, MEC supports modern 5G applications and mitigates traffic burdens, as well as lowering bottlenecks in backhaul networks. As a result, MEC efficiently supports IoT devices in task offloading, particularly for heavy and latency-sensitive tasks [9,10].

In spite of the substantial characteristics and potential of MEC, there have been several issues and challenges in relation to the MEC network, as follows. First, the storage capacities of edge servers are limited. This causes unbalanced loads and congestion among edge servers when numerous requests from IoT devices arrive. Second, because of the randomness and changeability of the networks, along with the long execution time of intensive applications in edge servers, the task failure rate due to offloading significantly rises [11,12]. Third, assigning resource allocations for task offloading, as well as developing effective and accurate offloading decision methods, are critical challenges in MEC. Furthermore, in heterogeneous IoT environments, the incoming streams of offloaded tasks from delay-sensitive and intensive applications need to be flexibly processed. Several approaches to resolving these issues are discussed below.

**Figure 1:**
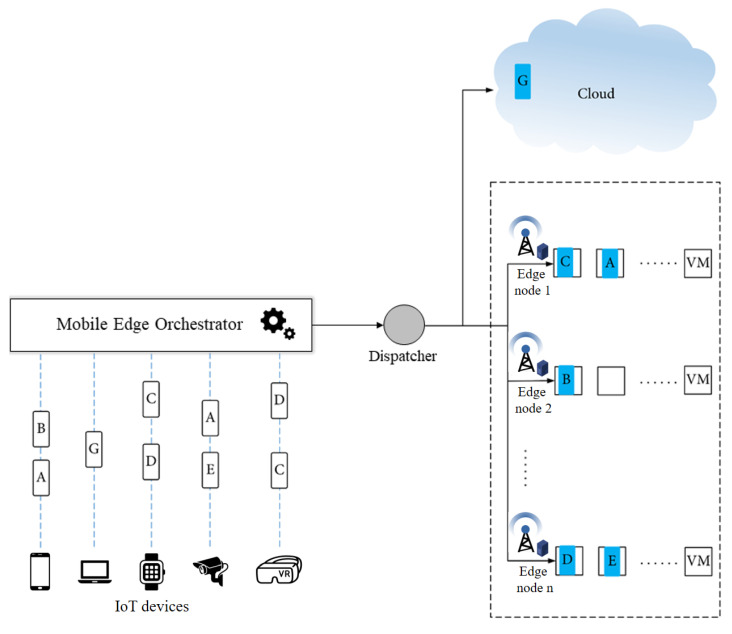
The flow of offloaded tasks through a mobile edge orchestrator.

One such approach is known as the mobile edge orchestrator (MEO), which is an orchestration embedded in MEC and which has been defined by ETSI [13]. The MEO has a broad perspective of the edge computing system and manages the available computing resources, network conditions, and the properties of the application [14,15]. Moreover, the MEO selects the appropriate mobile edge hosts for processing the applications based on constraints such as latency and inspects the available capacities of virtualization infrastructures for resource allocation [14]. Specifically, the MEO takes responsibility as a decision-maker in the network. Therefore, the target servers (e.g., the local edge server, the cloud server, and the remote edge server) for task offloading are efficiently decided by the MEO. The flow of offloaded tasks through an MEO and a dispatcher is illustrated in Figure 1. By achieving the topology of the network and analyzing the constraints, the MEO chooses the suitable target servers to execute tasks in the virtual machines (VMs) of the corresponding edge servers.

Recently, fuzzy logic has emerged as a reasonable alternative to handle orchestration issues such as task offloading and resource allocation in edge computing networks. Edge computing networks, such as fog computing and cloudlet computing in general, and the MEC network in particular, are types of swiftly changing uncertain networks. Fuzzy logic is appropriate to cope with changes of parameters, for instance, central unit processing (CPU) utilization on a VM, which regularly changes based on the number of tasks being executed or the bandwidth fluctuations that frequently occur when the number of users increases [16,17]. The reasons for this are briefly described as follows. First, because the fuzzy-logic-based approach has a lower computational complexity than other decision-making algorithms, it is effective for solving online and real-time problems without the need for detailed mathematical models [18]. Second, to support the heterogeneity of devices and the unpredictability of environments, fuzzy logic sets the rules, which are based on well-understood principles and the use of imprecise information provided in a high-level human-understandable format [16], and takes multiple network parameters of the network (e.g., task size, network latency, and server computational resources) into consideration [19]. Third, fuzzy logic considers multi-criteria decision analysis to determine the suitable servers at which IoT devices should offload the tasks [20]. Therefore, fuzzy-assisted MEO usefully supports task offloading by deciding where to offload the incoming requests from clients. There have been considerable works studying task offloading using the fuzzy logic approach. The authors of [21] proposed a cooperative fuzzy-based task offloading scheme for mobile devices, edge servers, and a cloud server in the MEC network. The authors of [22] proposed a fuzzy-based multi-criteria decision-making problem regarding the appropriate selection of security services. A novel fuzzy-logic-based task offloading collaboration among user devices, edge servers, and a centralized cloud server for an MEC small-cell network was studied in [23]. Nevertheless, these studies did not study how to find the best neighboring edge server to which the user device should offload the task, particularly when the network is crowded with a lot of IoT devices sending requests.

On the other hand, machine learning (ML) methods have been extensively integrated into heterogeneous 5G networks [24]. Among the ML-based approaches, such as supervised learning, unsupervised learning, and reinforcement learning, the algorithm of reinforcement learning is highly appropriate for handling problems with dynamically changing systems in a wireless network [25]. Moreover, reinforcement learning has lately become a promising technique for making offloading decisions [26], as well as performing resource allocations [27], in real-time. Reinforcement learning supports the MEO in the selection of suitable resources for applications by means of its useful features, such as its ability to learn without input knowledge and sequential decision-making within an up-to-date environment [28]. Additionally, Q-Learning and state-action-reward-state-action (SARSA) are two commonly used model-free reinforcement learning techniques with different exploration policies and similar exploitation policies [29]. A comparison of learning algorithms, such as GQ, R learning, actor-critic, Q-learning, and SARSA, on the arcade learning environment in [30] has shown that SARSA performs the most effectively in gaining the reward in comparison to other learning algorithms. Furthermore, a number of studies [31,32,33,34] on task offloading using the Q-Learning and SARSA techniques have been conducted to enhance the overall performance of the system (i.e., latency and energy consumption minimization, utility and resource optimization). However, these studies did not take advantage of the benefits of using neighboring edge nodes to serve offloaded tasks when the local edge servers have run out of computational resources. In this work, we model an MEC environment as a multi-tier system corresponding to the communication networks at different capacities, such as a WAN, a metropolitan area network (MAN), and a local area network (LAN). The system comprises thousands of IoT devices that continuously send a dynamic flow of requests for offloading. The main novelty of our work is to consider the best neighboring edge servers in which computational resources are excess for task offloading. We take advantage of learning through the experiences of SARSA to find the best neighboring edge servers. As a result, the load balancing among the edge servers is balanced, and the number of failed tasks is significantly reduced when the system receives the dynamic flow of requests from IoT devices. Moreover, we consider the use of an MEO as a decision-maker for task offloading in our system. In SARSA learning, the MEO takes responsibility as an agent, which decides the action. The key contributions of this paper are summarized as follows:  

- We aim to improve the rate of successfully executing offloaded tasks and to minimize the processing latency by determining the server at which the task should be offloaded, such as a cloud server, local edge server, or the best neighboring edge server, via a decision-maker.
- We define the MEO as a decision-maker for flexible task offloading in the system. The MEO manages the topology of the network and decides where the task will be executed. The MEO performs allocations in the MAN of the network.
- A collaboration algorithm between the fuzzy logic and SARSA techniques is proposed for optimizing the offloading decisions, which we call the Fu-SARSA algorithm. Fu-SARSA includes two phases: (i) the fuzzy logic phase and (ii) the SARSA phase. The fuzzy logic phase determines whether the task should be offloaded to a cloud server, local edge server, or neighboring edge server. If the MEO chooses the neighboring edge server to execute that task, the choice of the best neighboring edge server is considered in the SARSA phase.
- To model the incoming task requests, we consider four groups of applications: healthcare, AR, infotainment, and compute-intensive applications. They have dissimilar characteristics, such as their task length, delay sensitivity, and resource consumption. We compare and evaluate the results with four opponent algorithms, considering typical performance aspects such as the rate of task failure, service time, and VM utilization.
- Performance evaluations demonstrate the effectiveness of Fu-SARSA, which showed better results compared to the other algorithms.  

We have organized the rest of this paper as follows. Section 2 lists the related work on task offloading. In Section 3, we introduce the model of our proposed system and an overview of the Fu-SARSA algorithm. We briefly describe the first and second phases of the Fu-SARSA algorithm (i.e., the fuzzy logic phase in Section 4 and the SARSA phase in Section 5), respectively. Section 6 shows the simulation results of our proposal. Finally, we conclude the paper and discuss the future research approaches in Section 7.

## 2. Related Work

Task offloading and resource allocation are the key features in heterogeneous IoT networks. Based on the previous studies, the offloading decision can be classified into three main goals: minimizing the latency [35,36], minimizing the energy consumption [35,37,38,39,40], and maximizing the utility of the system [41,42,43]. The authors of [35] proposed an MEC-assisted task offloading technique to enhance latency and energy consumption by applying a hybrid approach: the grey wolf optimizer (GWO) and particle swarm optimization (PSO). Sub-carriers, power, and bandwidth were taken into consideration for offloading to minimize energy consumption. Shu et al. [36] introduced an efficient task offloading scheme to decrease the total completion time for processing IoT applications by jointly considering the dependency of sub-tasks and the contention between edge devices. In a study by Kuang et al. [37], using partial offloading scheduling and resource allocation, the energy consumption and total execution delay were optimized, while alsoconsidering the transmission power constraints in the MEC network. The offloading scheduling and task offloading decision issues were settled using the flow shop scheduling theory, whereas the suboptimal power allocation with partial offloading was achieved by applying the convex optimization method. Huynh et al. [38] formulated an optimization problem as a mixed-integer nonlinear program problem of NP-hard complexity to minimize the total energy consumption and task processing time in the MEC network. The original problem can be split into two subproblems: decisions of resource allocation and computation offloading, solved using a particle swarm optimization approach. By considering the total energy of both file transmission and task computing, the authors of [39] introduced an optimization problem for efficient task offloading to optimize the energy consumption in the MEC-enabled heterogeneous 5G network. Incorporating the various characteristics of the 5G heterogeneous network, an energy-efficient collaborative algorithm between radio resource allocation and computing offloading was designed. Khorsand et al. [40] formulated an efficient task offloading algorithm using the Technique for Order of Preference by Similarity to Ideal Solution (TOPSIS) and best-worst method (MWM) methodologies in order to determine the important cloud scheduling decision. The authors of [41] proposed joint task offloading and load balancing as a mixed-integer nonlinear optimization problem to maximize the system utility in a vehicular edge computing (VEC) environment. The optimization problem was divided into two subproblems—the VEC server selection problem, as well as the issues of the offloading ratio and computation resources. Lyu et al. [42] jointly optimized the heuristic offloading decision, communication resources, and computational resources to maximize the system utility while satisfying the QoS of the system. The study of Tran et al. [43] combined the resource allocation and task offloading decisions to maximize the system utility in the MEC network. To solve the task offloading problem, a novel heuristic algorithm was introduced to obtain the suboptimal solution in the polynomial time, whereas the convex and quasi-convex optimization methods were applied to handle the resource allocation problem. The task length for offloading can be classified into two categories: binary or full offloading [35,38,39,40,42,43] and partial offloading [36,37,41]. The tasks were either processed locally themselves or offloaded to the servers in a full offloading, whereas in partial offloading, a part of the task was locally processed and the other parts could be offloaded to the edge servers or a cloud server for execution.

To handle unpredictable environments with multi-criteria decision-making in IoT heterogeneous environments, fuzzy logic has been applied in recent research [21,23,44] to solve problems. Hossain et al. [23] proposed the fuzzy-assisted task offloading scheme among user devices, edge servers, and a cloud server in the MEC network, using one fuzzy logic stage with five fuzzy input variables. The authors of [21] proposed the cooperative task offloading mechanism among mobile devices, edge servers, and a cloud server, using eight fuzzy input parameters to achieve better performance with respect to processing time, VM utilization, WAN delay, and WLAN delay. Basic et al. [44] proposed an edge offloading and optimal node selection algorithm, applying fuzzy handoff control and considering several fuzzy crisp inputs, such as processor speed, bandwidth, and latency capabilities. To maximize the QoE of the system, An et al. [28] leveraged the advantages of both fuzzy logic and deep reinforcement learning Q-learning mechanisms for efficient task offloading in the vehicular fog computing environment. Moreover, deep learning methods, for example, in [28,29,45,46,47,48,49], have emerged as a potential means of efficient task offloading in modern networks. The authors of [45] formulated the computation offloading problem by jointly applying the reinforcement learning Q-learning approach and a deep neural network (DNN) to obtain the optimal policy and value functions for applications in the MEC network. Tang et al. [46] proposed a full task offloading scheme for delay-sensitive applications to minimize the expected long-term cost by combining three techniques, dueling deep Q-network (DQN), long short-term memory (LSTM), and double-DQN. The authors of [47] proposed a task offloading algorithm with low-latency communications, using the deep reinforcement learning technique to optimize the throughput of the user vehicles in highly dynamic vehicular networks. Jeong et al. [48] proposed a flexible task offloading decision method and took the time-varying channel into consideration to minimize the total latency of the application among edge servers in the MEC environment. To optimize the total processing time, a Markov decision process (MDP) technique was applied, and to handle the MDP problem, they designed a model-free reinforcement learning algorithm. To block the attacks from privacy attackers with prior knowledge, the authors of [49] proposed an offloading and privacy model to evaluate the energy and time consumption and privacy losses for intelligent autonomous transport systems. Taking the risk related to location privacy into consideration, a deep reinforcement learning method was applied and a privacy-oriented offloading policy was formalized to solve these problems. Alfakih et al. [29] applied the SARSA-based reinforcement learning method for task offloading and resource allocation to optimize the energy consumption and processing time of user devices in an MEC network. However, most of these studies did not consider the best neighboring edge server for task offloading in cases that all VMs in the local edge node are being used. Moreover, to ensure the QoS and QoE of the system, the task throw rate is considered in our study.

## 3. System Model and Overview of the Fu-SARSA Algorithm

### 3.1. System Model

**Figure 2:**
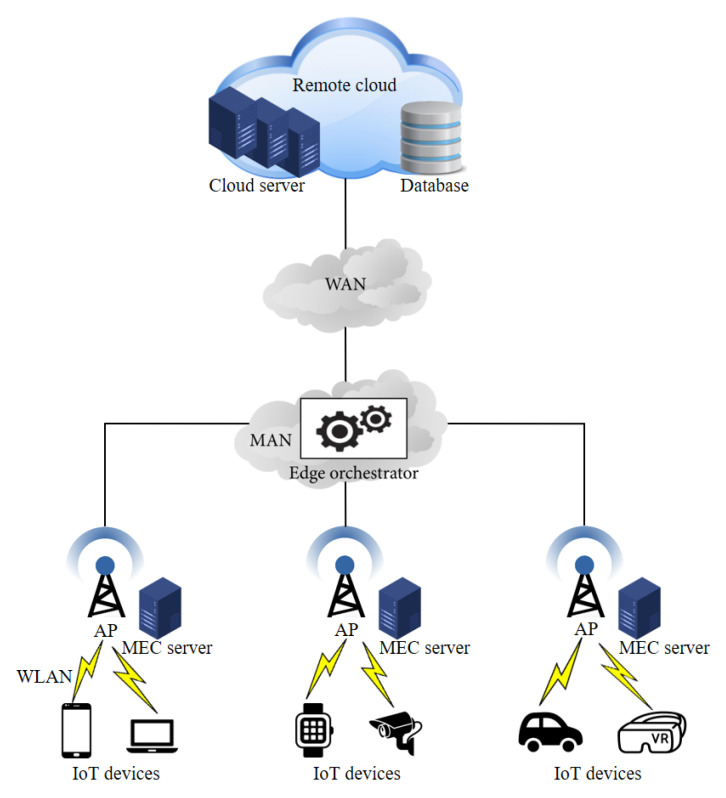
Proposed multi-tier MEC system architecture.

We propose an MEC network with three tiers: the IoT devices tier, the edges tier, and the centralized cloud tier. We depict the proposed system in Figure 2. The IoT devices represent the first tier, in which they send requests to edge servers for task offloading. The edge servers, together with a decision-maker, the MEO, are built in the second tier of the network. Devices connect to the edge servers via a wireless local area network (WLAN), whereas the edge servers are associated with an MAN. Moreover, the MEO is deployed as a decision-controller in the network center to handle applications’ offloading decisions. In this mechanism, a user can transfer the request to (i) a local edge server, which is the nearest edge server to the device (ii); a global cloud server, which has vast computation capabilities in the highest tier of the network; and (iii) a neighboring edge server (a.k.a., a remote edge server). In our scenario, the MEO chooses the best neighboring edge server to accomplish the application. Finally, the centralized global cloud server is involved in the third tier, which can be accessed through the WAN. We assume that the system includes *N* IoT devices and each device generates certain tasks. The task can be represented as $D_{i}≜\left(\kappa_{i},c_{i},T_{i}^{max}\right)$, where $\kappa_{i}$ denotes the size of the computation input data, $c_{i}$ is the computational resource requirement for task processing (i.e., the number of CPU cycles needed to compute one bit of the whole task), and $T_{i}^{max}$ denotes the maximum tolerable latency that is allowed to accomplish the task. Multiple processing tasks that exceed the limitation $T_{i}^{max}$ could lead to the congestion of the whole network. We assume that each MEC server owns a host, which runs a specific number of VMs. On the other hand, the storage capacity and computing resources in a centralized global cloud are much more powerful than those in an MEC server. The main goal of this research was to design an effective task offloading and resource allocation scheme in the MEC to reduce the overall latency and task failure rate. In this respect, we propose the Fu-SARSA algorithm, which is a task offloading decision algorithm, combining the fuzzy logic and SARSA techniques. We discuss Fu-SARSA in detail in Section 3.2 below.

### 3.2. Overview of Fu-SARSA Algorithm

**Figure 3:**
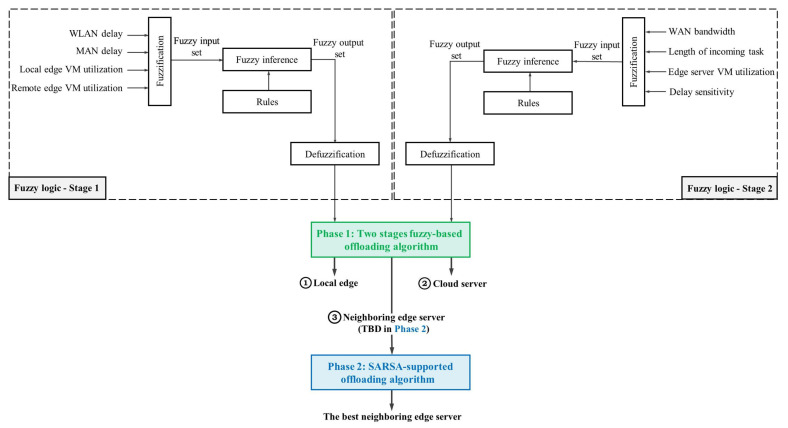
Fu-SARSA algorithm architecture.

In our study, we propose a flexible offloading decision algorithm, Fu-SARSA, the architecture of which is described in Figure 3. Fu-SARSA is divided into two phases: a fuzzy logic phase and SARSA phase. The fuzzy logic phase selects the server on which the task should be offloaded, such as a cloud server, local edge server, or neighboring edge server. If the MEO chooses the neighboring edge server to execute that task, the best neighboring edge server will be considered in the SARSA phase.

#### 3.2.1. Fuzzy Logic Phase

Due to the rapidly changing, uncertain nature of the MEC system, it is challenging to formulate accurate mathematical models [17,50]. Moreover, satisfying the multiple constraints of an optimization problem is not a straightforward problem in mathematical models; normally, a robust model cannot be detected because of the difficulty of the task [20]. Accordingly, it is not reasonable to apply the conventional offline optimization techniques since the number of incoming requests is not known in advance [20,23]. To settle these issues in unpredictable environments, the fuzzy-logic-enabled technique is a promising alternative. The main reason for this is that it easily handles the multi-criteria decision-making issue by analyzing multiple parameters under the same network [51,52]. The objective of our proposed two-stage fuzzy-based algorithm is to identify a target server for task offloading by examining different factors, for example, MAN delay, WAN bandwidth, length of the task, local edge VM utilization, remote edge VM utilization, and delay sensitivity.

To concisely describe how the first phase of Fu-SARSA works, we first explain the global fuzzy logic system (FLS). It is apparent that our proposed two-stage fuzzy-logic-based algorithm involves two FLSs. Both FLSs go through three main steps, fuzzification, fuzzy inference, and defuzzification, as shown in Figure 3. Specifically, a collection of reasonable IF-AND-THEN rules, followed by conclusions, is used to create the fuzzy rule set. In general, FLS maps the uncertain crisp input variables to a single value, the crisp output [53]. In the simple FLS, the crisp input variables have to go through three main operations: fuzzification, inference, and defuzzification. In our work, the crisp variables are properties of the network at a certain time, such as WAN bandwidth, task sensitivity, and average VM utilization, whereas the offloading decision is determined based on the value of the crisp output. We summarize the steps of the FLS as follows [20,53,54,55,56]:  

1. In the fuzzification step, the crisp input set is transformed into fuzzified sets. The crisp variable is mapped to the linguistic variable. The linguistic variable can be split into linguistic terms. The membership functions are used to determine each linguistic term’s value.
2. In the fuzzy inference step, the inference engine interprets the fuzzy input set based on the fuzzy rule collection to create the fuzzy output set.
3. In the final step, defuzzification obtains a single value from the fuzzy inference results. This process may be carried out by applying any defuzzification method.

Any type of crisp input variable can be considered in the fuzzy-logic-based system. In our study, we define the crisp variables that have significant impacts on the performance of the system. Therefore, these variables play a decisive role in the task offloading process. Each task will go through the FLS(s) to find a suitable place for offloading. According to the profile of the applications that create the demands, in the first FLS, we take the MAN delay, WLAN delay, local-edge VM utilization, and remote-edge VM utilization into consideration as the crisp input variables. Based on their parameters, the offloading decision for a task will be either the local edge server or the neighboring edge server. If the task decision opts for the local edge server, that task does not have to go through the second FLS step. Otherwise, the MEO can decide to offload the task to the cloud server or neighboring edge server in the second FLS step. First, we represent the importance of crisp input variables in the first FLS to the offloading decision, as follows [20]:

- WLAN delay: The parameter of WLAN delay needs to be considered, since the first tier of the network is covered by WLAN.
- MAN delay: To decide whether the task should be offloaded to the local edge server or the remote edge server, the parameter of MAN delay needs to be considered. If the MAN resources are packed due to the numerous requests to edge servers, the local edge server is more advantageous for offloading.
- Local edge VM utilization: The shortage of computational resources in the local edge server may cause offloading failure; therefore, local edge VM utilization is taken into account. Since the generated tasks are not evenly distributed, there must be some edge servers with excess resources, whereas the others have no resource capability for task processing. If the MAN capacity is comfortable, distributing the requests between the edge servers absolutely enhances the performance of the system.
- Remote edge VM utilization: If neighboring edge VM utilization is available and the local edge server capacity is used up, the neighboring edge sever should be the target server for offloading the task if the MAN delay is low.  

After the first FLS is completed, any tasks with offloading decisions with regard to the neighboring edge server have to go through the next FLS for a decision to made as to their execution at either a cloud server or any neighboring edge server. In addition, the WAN bandwidth, the VM resources of edge servers, and the characteristics of resource-intensive computational tasks play an important role in offloading decisions. Therefore, we consider four input variables for the second FLS: WAN bandwidth, the length of the task, the average VM utilization among edge servers, and the delay sensitivity of the task, which are briefly described as follows:  

- WAN bandwidth: To decide whether the task should be offloaded to the cloud server or not, the WAN bandwidth is a key variable that has to be considered. If the WAN communication delay is higher than the QoS requirement of the task or the network is too overloaded to cause data losses, the offloading decision should send the task to an edge server, rather than to the cloud server.
- Average VM utilization: This variable represents the mean utilization of all VMs running on servers in the network. Therefore, the remaining computational resources among edge servers can be calculated. If the utilization is above a certain threshold, the edge servers are considered packed due to the high number of offloaded tasks. Consequently, there is no better server than the powerful cloud server for offloading the task.
- Size of the task: The service time is determined based on the length of the tasks. The task length needs to be analyzed as a metric for offloading decisions. A heavy task should be transferred to a powerful cloud server to mitigate the resource burden among edge servers. In our work, the task length depends on the type of application. In the majority of cases, a 30 giga instructions (GI) compute-intensive application should be processed in a cloud server, whereas a 6 GI healthcare application is likely to be executed in an edge server.
- Delay sensitivity of the task: This variable refers to the tolerance of the task as it may take a longer time to execute due to network congestion or server utilization levels. The delay sensitivity of the request is determined by the application parameters. 

We briefly explain the two-stage fuzzy-logic-based task offloading algorithm in Section 4.

#### 3.2.2. SARSA Phase

Reinforcement learning is one of three broad categories of machine learning, which takes appropriate actions to maximize the total cumulative reward of the agent in certain states. Unlike supervised learning, instead of learning from a training set with answer keys, the model is trained to yield the desired output, whereas the reinforcement learning agent decides on a suitable action to perform the requests. In other words, the agent manages to learn from its experience without the training dataset [57]. Reinforcement learning uses reward and punishment as signs of positive and negative actions. Reinforcement learning consists of two main entities: the agent and the environment. The agent determines the appropriate action at a specific state of the environment, whereas the environment is the specific world with which the agent interacts. Based on the action obtained from the policy, the environment rewards the agent for the action performed and generates the next state.

Q-learning and SARSA are two common temporal difference (TD) reinforcement learning algorithms. The Q-value functions of the Q-learning and SARSA algorithms are formulated in Formulas (1) and (2), respectively.

(1) $$Q\left(s_{t},a_{t}\right)\leftarrow Q\left(s_{t},a_{t}\right)+\alpha \left[r_{t}+\gamma \max_{a}Q\left(s_{t+1},a\right)-Q\left(s_{t},a_{t}\right)\right]$$

(2) $$Q\left(s_{t},a_{t}\right)\leftarrow Q\left(s_{t},a_{t}\right)+\alpha \left[r_{t}+\gamma Q\left(s_{t+1},a_{t+1}\right)-Q\left(s_{t},a_{t}\right)\right]$$

where $s_{t}$ and $a_{t}$ are the state and action at the time *t*, $s_{t+1}$ and $a_{t+1}$ are the state and action at the time t+1, $r_{t}$ is the reward value, α is the learning rate, and γ is the discount factor.

Q-learning is an off-policy technique, whereas SARSA is an on-policy technique. Policy π specifies an action *a* that is taken in a state *s*. More precisely, π is a probability $\pi (a_{t}|s_{t})$ that an action *a* is taken in a state *s*. In off-policy learning, the Q-value function is achieved through performing actions (e.g., random actions). The Q-Learning algorithm chooses the best action *a* among a set of actions, whereas the action in the SARSA algorithm is taken according to policy π, for example, the epsilon-greedy (ε-greedy) policy. Since the learning is dependent on the current action by the current policy, SARSA is considered an on-policy learning technique. A comparison of Q-Learning and SARSA is briefly represented in Table 1.

**Figure 4:**
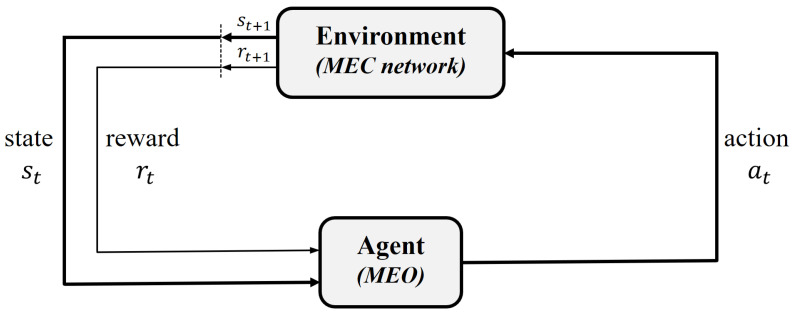
Agent–environment interaction in the network.

Reinforcement learning supports the software agents in intelligently deciding upon the best action in a particular state of the environment, in order to maximize their performance. Therefore, in this phase of our proposed algorithm, we determine the best neighboring edge server for task offloading using one of the reinforcement learning-based methods, that is, SARSA learning. The agent–environment interaction in our system is described in Figure 4. The MEO takes responsibility as a decision-maker in the MEC network. When the MEO receives the request from a task for an offloading decision, it is considered an agent which needs to choose the best offloading action for that task. The task offloading scenario occurs in the IoT heterogeneous MEC network; therefore, the parameters in the MEC refer to the environment for the agent. We precisely describe the SARSA-supported task offloading algorithm in Section 5.

## 4. Two-Stage Fuzzy-Logic-Based Task Offloading Algorithm

In this section, we briefly present the three main steps of the fuzzy reasoning mechanism: fuzzification, inference, and defuzzification. In an FLS, an inference engine works with fuzzy rules. An FLS maps crisp input variables to a crisp output using the theory of fuzzy sets [58]. In our work, we use the remote edge VM utilization, local edge VM utilization, WLAN delay, and MAN delay as crisp input variables for the first FLS, whereas the WAN bandwidth, average VM utilization among edge servers, task size, and delay sensitivity of the incoming task are the crisp input variables used for the second FLS. Based on the crisp outputs in both FLSs, the MEO chooses a suitable server, either a local edge server, a global cloud server, or the best remote edge server for an incoming task.

### 4.1. Fuzzification

Fuzzification is the conversion procedure that maps a crisp input value to a fuzzy value using the membership functions. Both FLSs in our work follow the same fuzzy logic steps, with four crisp input variables. We operate on input variables in the first and second FLS, defined as follows:

(3) $$F_{1}=\left\{\rho ,\eta ,\delta ,\varphi \right\}$$

where ρ is the WLAN delay, η is the MAN delay, δ is the local edge VM utilization, and φ is the neighboring edge VM utilization.

(4) $$F_{2}=\left\{\sigma ,\upsilon ,\kappa ,\tau \right\}$$

where σ is the WAN bandwidth, υ denotes the amount of VM resources that are being used on the edge server, κ is the length of the incoming task, and τ is the delay sensitivity of the incoming task.

An FLS uses non-numerical linguistic variables originating from natural language, rather than using numerical values. Linguistic variables use different linguistic values for each indicator. We use low (L), medium (M), and  high (H) as linguistic values for crisp input variables ρ, η, δ, φ, σ, υ, and τ. For the length of the incoming task κ, we define light (L), normal (N), and heavy (H) as linguistic values. The delay sensitivity of the task τ is evaluated by any real number from 0 to 1; therefore, this crisp input variable does not have the base unit. We describe the crisp input variables of both FLSs in Table 2 and Table 3.

During both the fuzzification and defuzzification FLS steps, membership functions are utilized. A set of membership functions for each variable is defined to fuzzify the crisp input into fuzzy linguistic terms. The membership function can be presented in a variety of forms, such as trapezoidal, left/right-shoulder, Gaussian, piecewise linear, triangular, singleton, and sigmoid forms [53]. In our study, we use the combination of triangular and left/right shoulder forms to represent the membership functions. The value of the membership function could be any real number between 0 and 1. We define the formulas of each type of membership function as follows:   

(5) $$\mu^{triangular}\left(x\right)=\left\{\begin{array}{cc} 0\; & if\;x⩽a\\ \frac{x-a}{b-a}\; & if\;a<x⩽b\\ \frac{c-x}{c-b}\; & if\;b<x<c\\ 0\; & if\;x⩾c \end{array}\right.$$

(6) $$\mu^{left-shoulder}\left(x\right)=\left\{\begin{array}{cc} 1\; & if\;x<a\\ \frac{b-x}{b-a}\; & if\;a<x<b\\ 0\; & if\;x⩾b \end{array}\right.$$

(7) $$\mu^{right-shoulder}\left(x\right)=\left\{\begin{array}{cc} 0\; & if\;x⩽a\\ \frac{x-a}{b-a}\; & if\;a<x<b\\ 1\; & if\;x⩾b \end{array}\right.$$

By using a set of membership functions, the fuzzifier determines the fuzzy terms for each input variable (WAN bandwidth, average VM utilization, length of the task, and delay sensitivity of the task), given as follows.

(8) $$F_{\sigma }\left(x\right)=\left[\mu_{\sigma }^{L}\left(x\right),\mu_{\sigma }^{M}\left(x\right),\mu_{\sigma }^{H}\left(x\right)\right]$$

(9) $$F_{\upsilon }\left(x\right)=\left[\mu_{\upsilon }^{L}\left(x\right),\mu_{\upsilon }^{M}\left(x\right),\mu_{\upsilon }^{H}\left(x\right)\right]$$

(10) $$F_{\kappa }\left(x\right)=\left[\mu_{\kappa }^{L}\left(x\right),\mu_{\kappa }^{N}\left(x\right),\mu_{\kappa }^{H}\left(x\right)\right]$$

(11) $$F_{\tau }\left(x\right)=\left[\mu_{\tau }^{L}\left(x\right),\mu_{\tau }^{M}\left(x\right),\mu_{\tau }^{H}\left(x\right)\right]$$

**Figure 5:**
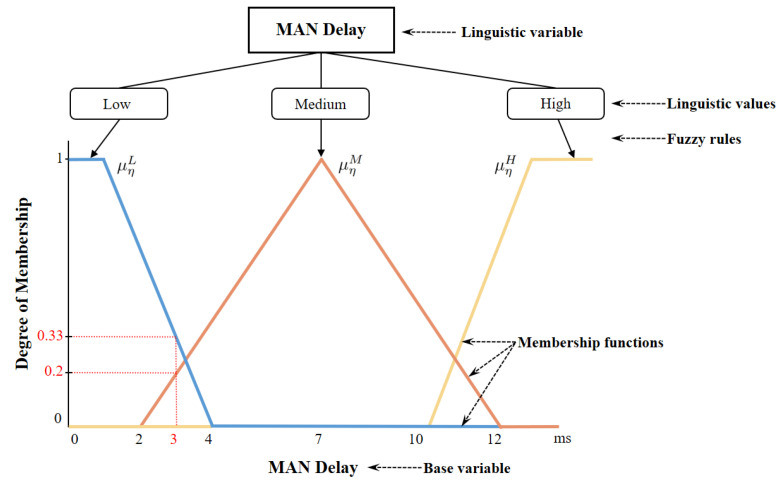
Example of a linguistic variable and its related components.

**Figure 6:**
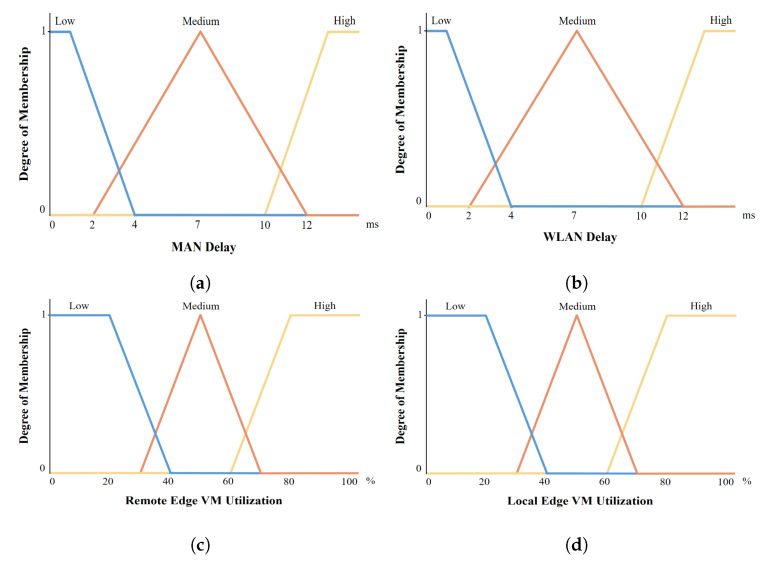
Set of membership functions for each input variable in the first FLS: (**a**) WLAN delay. (**b**) MAN delay. (**c**) Local edge VM utilization. (**d**) Remote edge VM utilization.

**Figure 7:**
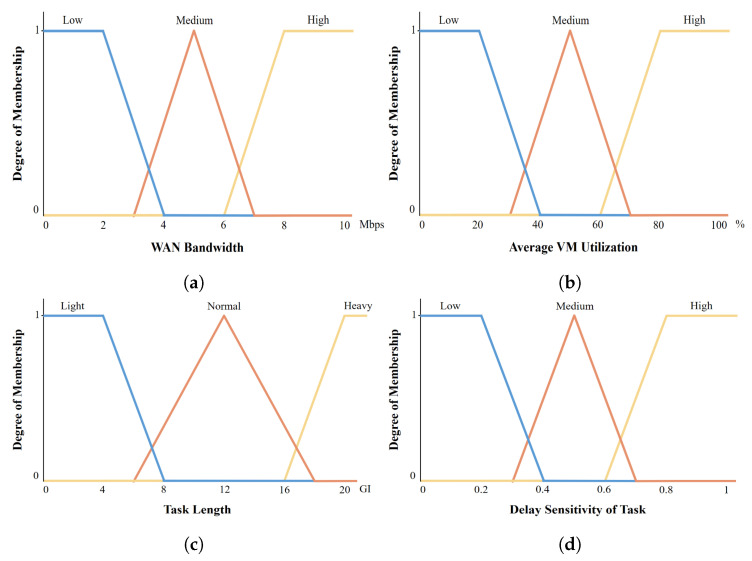
Set of membership functions for each input variable in the second FLS: (**a**) WAN bandwidth. (**b**) Average VM utilization. (**c**) Task length. (**d**) Delay sensitivity of the task.

An example of a linguistic variable with linguistic values and a set of membership functions—specifically, the variable of MAN delay—is depicted in Figure 5. As an example, we assume that the crisp input parameter of MAN delay η is 3 ms. The value of the left-shoulder membership function is $\mu_{\eta }^{L}\left(x\right)=\frac{4-3}{4-1}=\frac{1}{3}$. To calculate the value of $\mu_{\eta }^{L}\left(x\right)$, we determine the range value of crisp variables based on the range column in Table 2 and apply the Formula (6). Therefore, the values of a, x, and b will be 1, 3, and 4, respectively. As a<x<b, the value of $\mu_{\eta }^{L}\left(x\right)$ is equal to $\frac{b-x}{b-a}$. Similarly, the values of a, x, and b for the right-shoulder membership function are 10, 3, and 13. Since x<a, $\mu_{\eta }^{H}\left(x\right)=0$. The last membership function $\mu_{\eta }^{M}\left(x\right)$ has a triangular shape. We use the values of x, a, b, and c (i.e., 3, 2, 7, and 12) to calculate the value of $\mu_{\eta }^{M}\left(x\right)$, which is equal to 0.2. As a result, the values of membership functions with corresponding low, normal, and high linguistic values are calculated, which are $\frac{1}{3}$, 0.2, 0, respectively, and $F_{\eta }\left(x\right)=\left[\frac{1}{3},0.2,0\right]$. We define the set of membership functions as each input variable of the first and second FLSs in Figure 6 and Figure 7.

### 4.2. Fuzzy Inference

Fuzzy inference is an important process that maps the fuzzified inputs to fuzzy outputs using a set of fuzzy logic rules. The fuzzy rule base contains a series of IF-AND-THEN rules, followed by the conclusions [53]. Different linguistic variables are involved in each fuzzy rule. For instance, IF the average VM utilization is low AND the delay sensitivity of the task is low, THEN the task is offloaded to a remote edge server. To determine the fuzzy rules, we vary and use empirically fuzzy rule sets and choose the best rule combinations via computational experiments [20,23]. The number of fuzzy rules is $n=3^{4}=81$, since there are four crisp input variables with three linguistic values in each FLS of our proposed system. Therefore, a total of 162 rules are applied to the fuzzy inference system of the whole system. Table 4 shows an example of empirically fuzzy rules for the placement problem. Since both FLSs work in the same manner, we take the second FLS as an example.

Normally, in inference steps, the activation, aggregation, and accumulation methods are applied. In the aggregation step (a.k.a., the combination step), we apply minimum and maximum functions for AND and OR operators [20,59]. The activation step determines how the evaluated result of the IF part is utilized to the THEN part. Among the most commonly used activation operators, such as minimum and product, we chose the minimum function in our work [20,60,61]. Lastly, the accumulation method defines the combination of the multiple rules in a set of rules [60] by using maximum or minimum functions. In our work, we use the maximum function. In sum, the fuzzy inference system maps fuzzy input values to a fuzzy output value through steps using three different methods (i.e., aggregation, activation, and accumulation). The fuzzy output values $\mu_{cloud}$ and $\mu_{remoteedge}$ for offloading decisions, defined using the maximum function in the accumulation method, are given as follows.   

(12) $$\mu_{cloud}=max\left\{\mu_{cloud}^{R1},\mu_{cloud}^{R2},\ldots ,\mu_{cloud}^{R_{n}}\right\}$$

(13) $$\mu_{remoteedge}=max\left\{\mu_{remoteedge}^{R1},\mu_{remoteedge}^{R2},\ldots ,\mu_{remoteedge}^{R_{n}}\right\}$$

The general formula of the minimum function in the aggregation and activation methods is given as follows.

(14) $$\mu_{i}^{R_{n}}=min\left\{\mu_{\sigma }^{L_{\sigma }}\left(p\right),\mu_{\upsilon }^{L_{\upsilon }}\left(q\right),\mu_{\kappa }^{L_{\kappa }}\left(r\right),\mu_{\tau }^{L_{\tau }}\left(s\right)\right\}$$

where *i* refers to offloading decisions either to the cloud server or the remote edge server. The variables *p*, *q*, *r*, and *s* represent the crisp input parameters of crisp input variables σ, υ, κ, and τ, respectively. $L_{\sigma }$, $L_{\upsilon }$, $L_{\kappa }$, and $L_{\tau }$ are linguistic values of σ, υ, κ, and τ in the rule $R_{n}$, respectively.

We explain the fuzzy inference process by using an example. We assume that only five rules are defined, as shown in Table 4 and the values of the WAN bandwidth, average VM utilization, task length, and delay sensitivity of the task values are 8 Mbps, 70%, 17 GI, and 0.9, respectively. We analyze the fuzzy output $\mu_{cloud}$ in this example, so rules from R1 to R3 are considered. The minimum functions utilized in the aggregation and activation steps are as follows:

$$\mu_{cloud}^{R_{1}}=min\left\{\mu_{\sigma }^{M}\left(8\right),\mu_{\upsilon }^{H}\left(70\right),\mu_{\kappa }^{N}\left(17\right),\mu_{\tau }^{H}\left(0.9\right)\right\}$$

$$\mu_{cloud}^{R_{2}}=min\left\{\mu_{\sigma }^{H}\left(8\right),\mu_{\upsilon }^{M}\left(70\right),\mu_{\kappa }^{H}\left(17\right),\mu_{\tau }^{L}\left(0.9\right)\right\}$$

$$\mu_{cloud}^{R_{3}}=min\left\{\mu_{\sigma }^{H}\left(8\right),\mu_{\upsilon }^{H}\left(70\right),\mu_{\kappa }^{H}\left(17\right),\mu_{\tau }^{H}\left(0.9\right)\right\}$$

We can then obtain the value of fuzzy output $\mu_{cloud}$ based on $\mu_{cloud}^{R_{1}}$, $\mu_{cloud}^{R_{2}}$, $\mu_{cloud}^{R_{3}}$ using the maximum function:

$$\mu_{cloud}^{R_{1}}=min\left\{0,0.5,0.167,1\right\}=0$$

$$\mu_{cloud}^{R_{2}}=min\left\{1,0,0.25,0\right\}=0$$

$$\mu_{cloud}^{R_{3}}=min\left\{1,0.5,0.25,1\right\}=0.25$$

$$\mu_{cloud}=max\left\{\mu_{cloud}^{R_{1}},\mu_{cloud}^{R_{2}},\mu_{cloud}^{R_{3}}\right\}=max\left\{0,0,0.25\right\}=0.25$$

### 4.3. Defuzzification

The last step in FLS is the defuzzification step, which converts the fuzzy output obtained by the fuzzy inference system to a single crisp value. Centroid defuzzification is applied in the defuzzification step, which determines the center of the inferred fuzzy outputs. Several methods are utilized for centroid calculation, such as the weighted fuzzy mean (WFM), mean of maximum (MOM), fuzzy clustering defuzzification (FCD), and center of gravity (COG) methods [62]. In our work, we use the COG method, which returns the center of gravity under the curve [53]. Mathematically, it is obtained as follows:

(15) $$\omega =\frac{\int x\mu (x)dx}{\mu (x)dx}$$

After the calculation is complete, the crisp output value ω is obtained, which has a value between 0 and 100. Since our proposed algorithm has two FLSs, we define the impact of two crisp outputs $\omega_{1}$ and $\omega_{2}$ on offloading decisions as follows.

(16) $$Decision_{1}=\left\{\begin{array}{c} \;\mathrm{local}\;\mathrm{edge}\;\mathrm{server},\;\omega_{1}<50\\ \;\mathrm{candidate}\;\mathrm{edge}\;\mathrm{server},\;otherwise \end{array}\right.$$

(17) $$Decision_{2}=\left\{\begin{array}{c} \;\mathrm{remote}\;\mathrm{edge}\;\mathrm{server},\;\omega_{2}<50\\ \;\mathrm{cloud}\;\mathrm{server},\;otherwise \end{array}\right.$$

**Figure 8:**
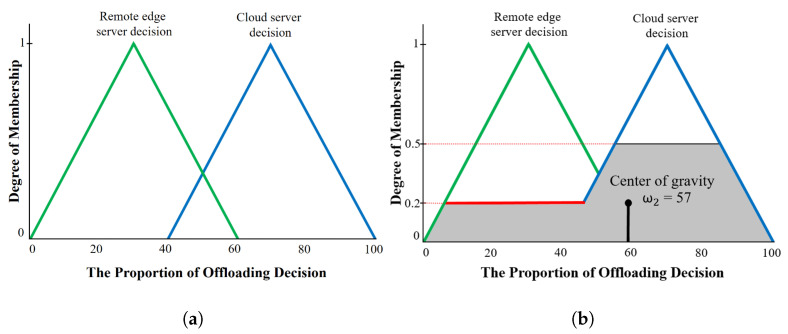
Defuzzification: (**a**) Membership functions of fuzzy outputs. (**b**) Crisp output calculation using COG.

The membership function for offloading decisions is shown in Figure 8a. We assume that the values of inferred fuzzy outputs $\mu_{cloud}$ and $\mu_{remoteedge}$ are 0.5 and 0.2. Applying Equation (15), the result of crisp output $\omega_{2}$ will be approximately 57, as shown in Figure 8b. Based on the value of the output result, the MEO decides to send the task to the cloud.

In conclusion, we briefly represent the first phase of our proposed algorithm Fu-SARSA, which is the two-stage fuzzy-logic-based task offloading algorithm in Algorithm 1 with the time complexity O(1) and space complexity O(1).
**Algorithm** **1** Two-Stage Fuzzy-Logic-Based Task Offloading Algorithm**Input:** The incoming task, T**Output:** Offloading decision target, O1:The first FLS starts;2:MEO reads the network topology (ρ,η,δ,φ);3:Calculate the crisp output $\omega_{1}$← Equation (15);4:**if** $\omega_{1}$ < 50 **then**5: O ← Local edge server;6: The first FLS ends;7:**else**8: The first FLS ends; the second FLS starts;9: MEO reads the network topology (σ,υ,κ,τ);10: Calculate the crisp output $\omega_{2}$← Equation (15);11: **if** $\omega_{2}$ < 50 **then**12:  O ← Neighboring edge server;13: **else**14:  O ← Cloud server;15: **end if**16: The second FLS ends;17:**end if**18:**return** O;

## 5. SARSA-Supported Task Offloading Algorithm

In the second phase of our proposed algorithm, Fu-SARSA, we apply the SARSA reinforcement learning algorithm to find the best neighboring edge servers for task offloading. The MEO decides on the edge server used for processing a task by considering the environment of the network, based on the MAN parameters. We define the transmission model and computation model in Section 5.1 and apply the SARSA-based reinforcement learning algorithm to support the task offloading in Section 5.1.

### 5.1. Communication Model and Computation Model

We define $t_{i}^{jk}$ as representing the time it takes to transmit the requested task from the local edge server *j* connecting to the IoT device *i* to the neighboring edge server *k*. The task transmission time $t_{i}^{jk}$ is formulated as follows:

(18) $$t_{i}^{jk}=\frac{\kappa_{i}}{R_{MAN}}+t_{MAN}^{jk}$$

where $\kappa_{i}$ is the size of the input data and $R_{MAN}$ is the transmission rate in the MAN. The MAN propagation delay is defined as follows:

(19) $$t_{MAN}^{jk}=\frac{d_{jk}}{s_{MAN}}$$

where $s_{MAN}$ is the wave propagation speed and $d_{jk}$ is the distance between the edge servers *j* and *k* and is calculated as $\sqrt{{\left(x_{j}-x_{k}\right)}^{2}+{\left(y_{j}-y_{k}\right)}^{2}}$. $\left(x_{j};y_{j}\right)$ and $\left(x_{k};y_{k}\right)$ are the coordinates of edge servers *j* and *k*, respectively.

Let $t_{k}^{i}$ denote the computation time of the task that was requested from IoT device *i* in the edge server *k*. $t_{k}^{i}$ is formulated as follows:

(20) $$t_{i}^{k}=\frac{c_{i}}{f_{i}^{k}}$$

where $c_{i}$ is the computational resources required for task processing (i.e., the number of CPU cycles needed to compute one bit of the whole task), and $f_{k}^{i}$ is the amount of computational resources that the edge server *k* assigns to the IoT device *i*.

In our model, the MEO will determine whether the tasks will be executed on the edge server, cloud server, or neighboring edge server. However, the best neighboring edge server is considered to minimize the latency of the task and to balance the load balancing among edge servers. The total processing delay of a task generated from the IoT device *i* is as follows:

(21) $$T_{i}^{jk}=t_{i}^{jk}+t_{i}^{k}$$

We aim to minimize the latency of each task while guaranteeing a stable balance among the edge servers, which are managed by the MEO. Define $x=\{x_{i}^{jk}\}$ as the vector of the neighboring server selection decision and $f=\{f_{ij}\}$ as the computational resource vector. In the MEC network scenario, *M* edge servers serve the offloading needs of *N* IoT devices. We formulate the optimization problem as follows:

$$\max_{x,f}\;\;\sum_{i=1}^{N}T_{i}$$

(22a) $$s.t.\;\;T_{i}\leq T_{i}^{max}\;$$

(22b) $$\;\;\;\;\;\;\;\sum_{j=0}^{M}x_{ij}=1\;\;$$

(22c) $$\;\;\;\;\;\;\;\;\;\;\;\;\;\;0\leq \sum_{i=1}^{N}f_{i}^{k}x_{i}^{jk}\leq F_{k}$$

(22d) $$\;\;\;\;\;\;\;\;R_{MAN}\geq R_{i}^{min}$$

(22e) $$\;\;\;\;x_{i}^{jk}\in \left\{0,1\right\}$$

The first Constraint (22a) guarantees that the processing time for a task cannot exceed the tolerable latency allowed to accomplish the task, $T_{i}^{max}$. Constraints (22b) and (22e) state that each device can offload the task to only one edge server. If the value of $x_{i}^{jk}$ equals zero, the task cannot be offloaded to the edge server *k*. Constraint (22c) ensures that the total computational resources assigned to all tasks on edge server *k* do not exceed the total computation resources of this server. Constraint (22d) ensures that the transmission rate of the IoT device *i* is greater than the minimum requirement of the task transmission rate.

The integer constraint $x_{i}^{jk}$ makes the optimization problem a mixed-integer, nonlinear programming problem, which is in general non-convex and NP-hard. Solving this problem using traditional algorithms has become challenging for high-complexity networks and the algorithms’ performance is closer to the bottleneck [63]. Therefore, we apply the SARSA-based reinforcement learning algorithm to settle the problem.

### 5.2. SARSA-Supported Offloading Decision

The term SARSA represents a quintuple $\left(s_{t},a_{t},r,s_{t+1},a_{t+1}\right)$, in which new actions and states are sequentially updated. In our system, the MEO receives the requests for task offloading. By reading and considering several important parameters of network topology at the current state $s_{t}$, the MEO agent decides on the best offloading action $a_{t}$ for the request. In SARSA, an ε-greedy policy is implemented to decide the action of the agent. The ε-greedy policy is a simple method to balance exploration and exploitation by randomly choosing between exploration and exploitation. Normally, the agent exploits most of the time in the case that ε is selected as a small number, close to zero. Therefore, in our work, the agent chooses the best action with a probability of 1−ε. As soon as an action is completed, the reward is achieved based on the reward function, and the new Q-value is updated. The Q-value is iteratively updated for each state–action pair using the Bellman equation until the Q-function converges to the optimal Q-function, $Q_{*}$. The Bellman Optimality equation is defined as below:

(23) $$Q_{*}\left(s,a\right)=E\left[r_{t}+\gamma \;q_{*}\left(s_{t+1},a_{t+1}\right)\right]$$

To ensure that the Q-value converges to an optimal Q-value $Q_{*}$, for the given state-action pair, the value of Q should be near the right-hand side of the Bellman equation. The new Q-value for the state–action pair at a certain time is defined as follows:

(24) $$Q_{*}\left(s,a\right)=\left(1-\alpha \right)q\left(s,a\right)+\alpha \left(r_{t}+\gamma q\left(s_{t+1},a_{t+1}\right)\right)$$

The state, action, and reward function of the SARSA-supported task offloading process are given below:

(1) State

At the beginning of the state transition, the properties of the environment are observed by the agent. The state of the environment is defined as follows:

(25) $$s_{i}\left(t\right)≜\left(\kappa_{i},\eta ,\upsilon ,\varphi_{k}^{M},\left(x_{k}^{M};y_{k}^{M}\right)\right)$$

where $\kappa_{i}$ is the size of the task, η is the MAN delay, υ is the average VM utilization of the edge servers, $\varphi_{k}^{M}$ represents the set of the neighboring VM utilization of each edge server k∈M, $\left(x_{k}^{M};y_{k}^{M}\right)$ contains the set of coordinates of each edge server k∈M.

(2) Action

For each time step *t*, the MEO decides on the task offloading action according to the ε-greedy policy. If the MEO chooses an action using the exploiting method, the task will be offloaded to the nearest neighboring edge server with excess computational resources. If the MEO chooses an action using the exploring method, the task is offloaded to the edge server with the lowest VM utilization. We define the actions as follows:

(26) $$a=\left\{\phi_{1},\phi_{2}\right\}$$

where the action $\phi_{1}$ offloads the task to the nearest neighboring edge server with the lowest VM utilization, and $\phi_{2}$ offloads the task to the edge server with the lowest VM utilization. In our study, we consider that the action $\phi_{1}$ is the best action. The agent uses a probability of 1−ε to select the best action.

(3) Design of the reward function

Following the agent-environment interaction during the state *t*, the agent receives feedback from the environment, that is, reward *r*, which reflects how effectively the agent learns from the environment. In a reinforcement learning algorithm, the reward function is normally designed based on the objectives of the system. We design a negative reward function to match the goals of our proposed system model, such as minimizing the task failure rate and the processing time of the task. The aim of the reward function is to evaluate the effectiveness of the actions of the agent. In fact, the value of the reward reaches the state of convergence after a certain episode. The faster the values of reward converge, the more effectively the agent learns from the environment. The values of the reward vary for different actions, defined as follows:

(27) $$r_{t}\left(s_{t},a_{t}\right)=-\lambda_{\phi }\left(d_{r}+T_{jk}^{i}\right)-\rho$$

where $d_{r}$ is the distance reward when the task is offloaded from the local edge server *j* to the neighboring edge server *k*, and $T_{jk}^{i}$ is the task processing delay. $\lambda_{\phi }$ denotes the satisfactory variable, which has a higher value if the agent chooses the best action. ρ is the penalty variable applied when the task is unsuccessfully offloaded. Let $d_{jk}$ denote the distance between edge servers *j* and *k*. As the edge servers are uniformly distributed in the network, we can calculate the distance between the two furthest edge servers, that is, $d_{max}$. The distance reward is formulated as follows:

(28) $$d_{r}={\left(\frac{d_{jk}}{d_{max}}\right)}^{\frac{1}{2}}$$

In this paper, we propose the SARSA-supported task offloading algorithm to obtain the best neighboring edge server for task offloading. The algorithm’s process is shown in the Algorithm 2 with time complexity O(T) and space complexity O(SAH), where *T* is the total number of steps performed by the agent, *S* is the number of states, *A* is the number of actions, and *H* is the number of steps per episode [64].
**Algorithm** **2** SARSA-Supported Task Offloading Algorithm**Input:** The incoming tasks for offloading**Output:** Efficient offloading decision with latency minimization1:Initialize the network parameters ← Equation (25);2:Initialize the Q-value. Set Q-value to 0;3:**for** episode = 1 **do** until convergence4: MEO reads the current network state *s*;5: Observe the state *s*;6: **for** each step in episode **do**7:  Select action $a=\left\{\phi_{1},\phi_{2}\right\}$ for task *i* using ε-greedy policy;8:  Take action a, calculate reward $r_{t}$;9:  $r_{t}\leftarrow$ Equation (27);10:  Observe next state $s_{t+1}$;11:  $s_{t+1}\leftarrow$ MEO reads the new state;12:  Update Q-value: $q^{new}\left(s,a\right)=\left(1-\alpha \right)q\left(s,a\right)+\alpha \left(r_{t}+\gamma q\left(s_{t+1},a_{t+1}\right)\right)$;13: **endfor**14:**endfor**

## 6. Performance Evaluation

For the network simulation, we used a realistic simulation that enabled multi-tier edge computing, named EdgeCloudSim [65]. To attain a more realistic simulation environment, EdgeCloudSim was used to perform an empirical study for the WLAN and WAN using real-life properties. Furthermore, the MAN delay was achieved using a single server queue model with Markov-modulated Poisson Process (MMPP) arrivals. The VM numbers per edge and cloud server were 8 and 4, respectively. The simulation parameters for the MEC network are briefly presented in Table 5.

To evaluate this proposal, we studied numerous scenarios with different numbers of IoT devices. Specifically, the minimum and the maximum number of IoT devices were 250 and 2500, respectively. The difference in IoT devices between the two consecutive scenarios was 250 devices. In a real-world generic edge computing environment, IoT devices generate various types of applications. However, to decide on the application types, we considered the most studied edge computing use cases in the recent research. We used four typical types of applications for more realistic simulations: healthcare, augmented reality (AR), infotainment, and compute-intensive applications. Specifically, first, a health application that uses a foot-mounted inertial sensor was studied to analyze users’ walking patterns in [66]. Second, the authors of [67] proposed an AR application on Google Glass, which is a head-mounted intelligent device that can be worn as wearable computing eyewear. Third, Guo et al. [68] proposed vehicular infotainment systems for driving safety, privacy protection, and security. Finally, to optimize the delay and energy consumption, compute-intensive services were proposed in [69]. The characteristics of these application types are described in Table 6. The use percentage of the application shows the portion of IoT devices generating this application. The incoming tasks were distributed over time based on the task interval indicator; for example, the MEO would receive a request for a healthcare application every 3 s. The delay sensitivity indicator measured the delay sensitivity of the task. The AR application was assumed to be a delay-sensitive application since its delay sensitivity value was 0.9, whereas the compute-intensive case was delay-tolerant with a very low delay sensitivity value of 0.15. The IoT devices implemented applications during the active period and rested during the idle period. The percentage of VM utilization on edge or cloud servers was subject to the length of the task.

**Figure 9:**
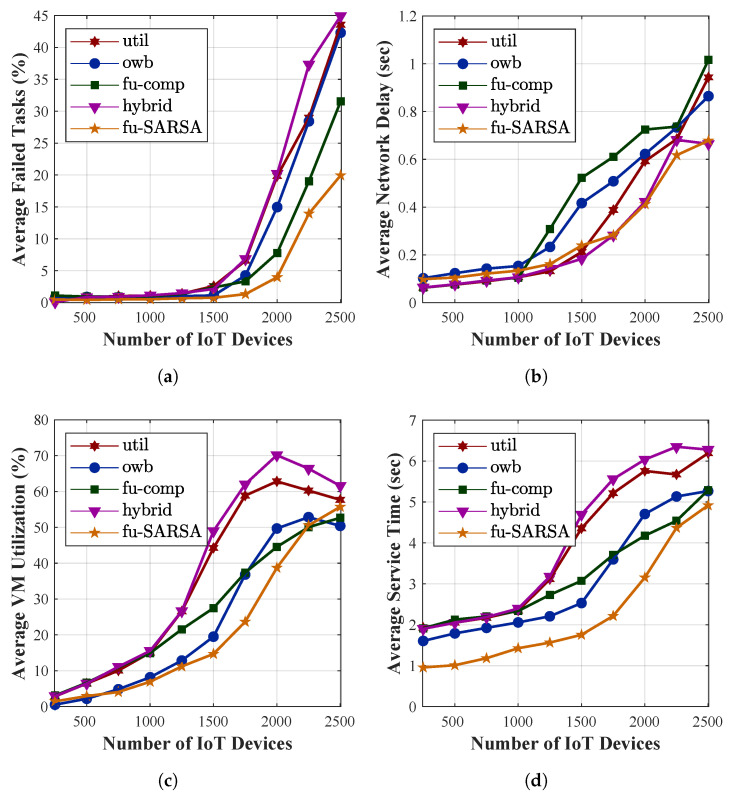
Comparative evaluations in terms of all application types: (**a**) Average task failure rate. (**b**) Average service time. (**c**) Average network delay. (**d**) Average VM utilization.

**Figure 10:**
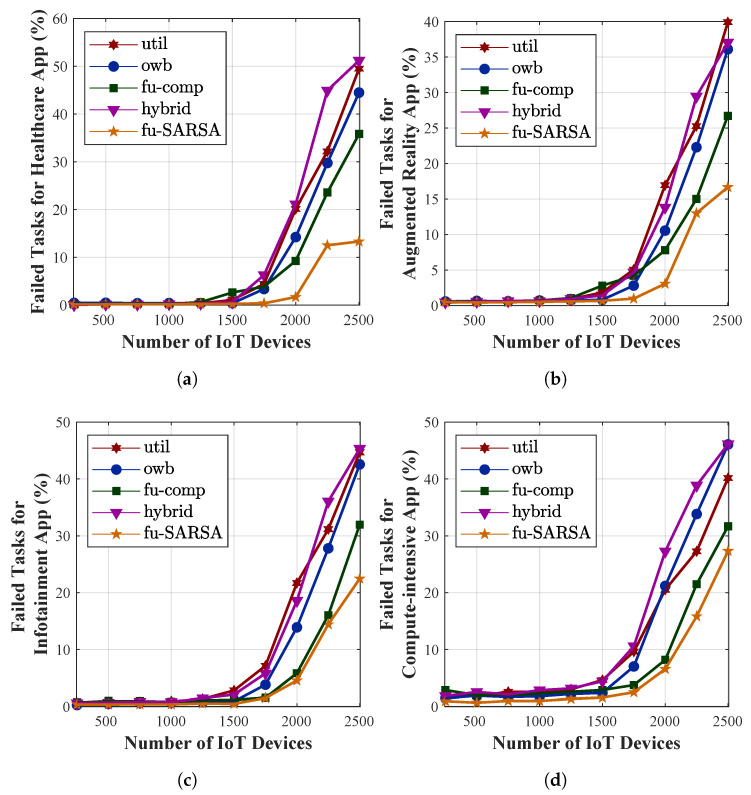
Comparison of task failure rate with different application types: (**a**) Healthcare application. (**b**) AR application. (**c**) Infotainment application. (**d**) Compute-intensive application.

**Figure 11:**
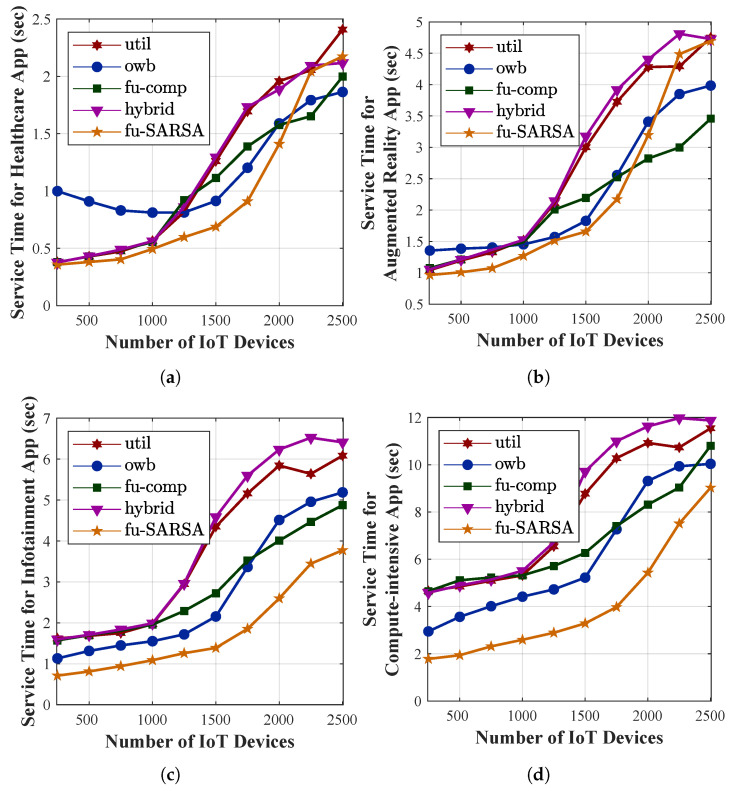
Comparison of service time with different application types: (**a**) Healthcare application. (**b**) AR application. (**c**) Infotainment application. (**d**) Compute-intensive application.

To verify its performance, we compared our proposal with other task-offloading approaches: utilization, online workload balancing (OWB), a fuzzy-based competitor, and a hybrid approach. The utilization approach depended on the local VM utilization threshold in the decision as to whether a task should be offloaded to either the remote edge server or a centralized cloud. By considering the lowest VM utilization of any edge server in the network, the OWB approach preferred to offload the task to these servers. The fuzzy-based competitor [70] utilized four crisp input variables (i.e., task length, network demand, delay sensitivity, and VM utilization) and one FLS process. In this approach, the task could be offloaded to one of the following three server types: a local edge server, a neighboring edge server, or a cloud server. Finally, the hybrid approach analyzed the WAN bandwidth and local VM utilization to offload the task to either the local edge server or a cloud server. In our work, we evaluated several criteria, such as the task failure rate, the percentages of VM utilization, the service time required for accomplishing the applications, and the network delay, in the results of our simulation. The utilization, OWB, fuzzy-based competitor, and hybrid approaches are abbreviated as util, owb, fu-comp, and hybrid, respectively, in the evaluation figures. We compared them to our proposal algorithm, Fu-SARSA, and study their performance. The performance of the main criteria in terms of the average results of all applications are compared in Figure 9. Moreover, the comparison of the task failure rate between the approaches with four different application types is depicted in Figure 10, whereas the comparison of service time for the different application types is represented in Figure 11. In this paper, we study each criterion on the basis of the simulation results.

The main aim of our proposed algorithm Fu-SARSA was to reduce the rate of task failure. The average percentage of failed tasks for all applications is presented in Figure 9a. The performances of all approaches were similar when the number of IoT devices was below 1500. The network became congested at 1750 IoT devices and reached the peak congestion between 2000 and 2500 devices. Compared to the other competitors, our proposal, Fu-SARSA, showed the best efficiency when the network was overloaded. Due to the network losses on MAN resources, many tasks were not successfully offloaded in the other approaches. The utilization and hybrid approaches exhibited the worst results because these techniques only consider the threshold of the MAN bandwidth or VM utilization. In the real-world 5G environment, applications need to adapt to flexible changes in network parameters. During the comparison with different application types, the failed task percentage of Fu-SARSA was much lower than that of any other algorithm. Particularly, Fu-SARSA worked best for healthcare and AR applications, as shown in Figure 10a,b, because these applications required a small CPU capacity to ensure that the load balancing among servers was stable when the Fu-SARSA algorithm was operated in the network. For the heavy tasks, such as the compute-intensive and infotainment applications, both Fu-SARSA and fuzzy-based competitor approaches showed good performance in reducing the task failure rate, as depicted in Figure 10c,d, especially for the scenario with 2500 IoT devices. Figure 9d represents the average VM utilization of edge servers. As many heavy tasks from 2500 devices were successfully offloaded using the Fu-SARSA approach, a larger amount of CPU resources was used to process the tasks.

Service time is an important criterion to evaluate the effectiveness of the system. In the heterogeneous 5G network, the service time required to accomplish the application should be as short as possible to satisfy the QoS of the system. Figure 9b represents the average service time for the tasks in terms of all types of applications. The service time of the task is the sum of the processing time and the network delay. Our proposal, Fu-SARSA, provided the best results in terms of the service time in comparison with the other approaches, because it took the network conditions and properties of the incoming task into account; hence, the best decisions, such as the choice of the best neighboring edge server, were made. When the system load was high and the number of IoT devices was more than 1250, the utilization and hybrid approaches showed poor performance. In the worst-case scenario, approximately 6 s were needed to accomplish the task. The OWB approach preferred to offload the task to the VM with the lowest resource capacity and the fuzzy-based competitor approach considered the network parameters for an offloading decision; therefore, they showed better results and processed the task faster. As a result, the average network delay of these algorithms was higher than that of other algorithms when the MAN and WAN resources became congested, as depicted in Figure 9c. Concerning the average service time required with different application types, Fu-SARSA worked well for heavy computational tasks, as shown in Figure 11c,d. However, more service time was needed for the healthcare and AR applications in cases with many IoT devices, for instance, from 2000 to 2500 devices. As explained above, Fu-SARSA worked the best for healthcare and AR applications, since it provided a significantly lower task failure rate, especially when the network was overloaded. The network delay for these applications was longer; therefore, the average service time was higher, as illustrated in Figure 11a,b.

**Figure 12:**
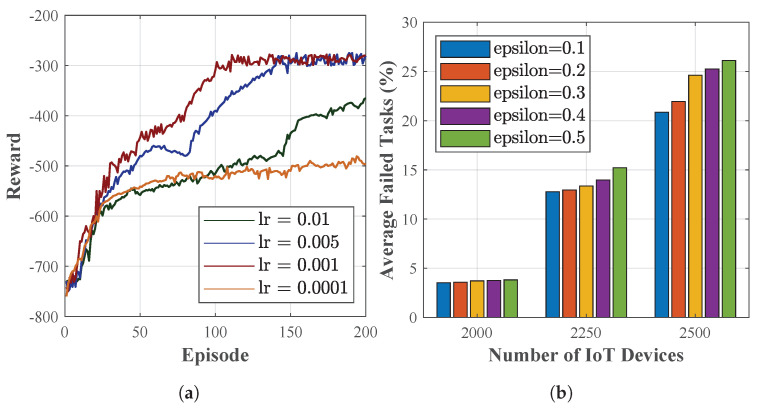
Study of variables in SARSA reinforcement learning: (**a**) Reward value through each episode with different learning rates. (**b**) Average task failure percentage with different epsilon values.

As the Fu-SARSA applies SARSA-based reinforcement learning, we investigated the convergence of the algorithm at some typical learning rates, abbreviated as lr in Figure 12a. The reward of the system was evaluated with respect to the order of episodes. Four learning rate values were taken into account: 0.01, 0.005, 0.001, and 0.0001. The algorithm worked best with a learning rate of 0.001, achieving the fastest convergence state in comparison to the other learning rates. When the learning rate was greater than 0.001, more episodes needed to be converged. As the learning rate decreased (i.e., lr = 0.0001), the performance worsened, with a longer time needed to reach stable values. On the other hand, to attain better offloading decisions with the on-policy reinforcement learning technique, the value of the epsilon variable should be considered. Since the agent chose the best action with a probability of 1−ε, epsilon was selected as a small number, close to 0. For this reason, we investigated five epsilon values of 0.1, 0.2, 0.3, 0.4, and 0.5 in terms of the average task failure percentage when the network was overloaded. Three scenarios, involving 2000, 2250, and 2500 IoT devices, were studied to evaluate the different epsilon values, as depicted in Figure 12b. Compared to other values, the epsilon value of 0.1 was the most effective in terms of the task offloading performance and was demonstrated to be compatible with the ε-greedy policy.

## 7. Conclusions

In this paper, we aimed to improve the service time and minimize the task failure rate in a heterogeneous MEC network by using an algorithm based on collaboration between fuzzy logic and SARSA learning. The IoT device offloads the task to an edge server, global cloud server, or best neighboring edge server under the management of an MEO decision-maker. To obtain more realistic results, we simulated the network using four typical applications—healthcare, AR, infotainment, and compute-intensive applications. Our proposed Fu-SARSA algorithm provided better results in comparison with other algorithms. Fu-SARSA worked best for healthcare and AR applications in terms of the failed task rate and service time, particularly in cases where the network was congested with numerous IoT devices. In the future, we will apply deep learning for efficient offloading to enhance system performance.

## Figures and Tables

**Table 1:** Q-Learning and SARSA comparison.

	Q-Learning	SARSA
Learning type	Off-Policy	On-Policy
Next action decision	Next action is determined based on the best action in a set of actions *a*	Next action is determined based on policy π (e.g., ε-greedy policy)
Q-table update rule	Updated based on the greedy policy from the Q-table	Updated based on the current state, current action, obtained reward, next state, and next action
Convergent	Converged to an optimal solution under the assumption that, after generating experience and training, the system switches over to the greedy policy	Converged to an optimal solution under the assumption that the system keeps following the same policy that is used to achieve the experience
Application cases	Preferable in situations where the agent’s performance is not considered during the training process, but switches to learn an optimal greedy policy eventually	Preferable in situations where an agent’s performance is taken into consideration during the process of learning and generating the experience
Popularity	More popular	Less popular

**Table 2:** Related parameters in the fuzzification of the first FLS.

Input Variable	Notation	Linguistic Value	Membership Function Type	Range
WLAN Delay (ms)	ρ	low	left-shoulder	0, 1, 4
		medium	triangular	2, 7, 12
		high	right-shoulder	10, 13, >13
MAN Delay (ms)	η	low	left-shoulder	0, 1, 4
		medium	triangular	2, 7, 12
		high	right-shoulder	10, 13, >13
Local edge VM utilization (%)	δ	low	left-shoulder	0, 20, 40
		medium	triangular	30, 50, 70
		high	right-shoulder	60, 80, >80
Remote edge VM utilization (%)	φ	low	left-shoulder	0, 20, 40
		medium	triangular	30, 50, 70
		high	right-shoulder	60, 80, >80

**Table 3:** Related parameters in the fuzzification of the second FLS.

Input Variable	Notation	Linguistic Value	Membership Function Type	Range
WAN bandwidth (Mbps)	σ	low	left-shoulder	0, 2, 4
		medium	triangular	3, 5, 7
		high	right-shoulder	6, 8, >8
Average VM utilization (%)	υ	low	left-shoulder	0, 20, 40
		medium	triangular	30, 50, 70
		high	right-shoulder	60, 80, >80
Task Length (GI)	κ	light	left-shoulder	0, 4, 8
		normal	triangular	6, 12, 18
		heavy	right-shoulder	16, 20, >20
Delay sensitivity of the task	τ	low	left-shoulder	0, 0.2, 0.4
		medium	triangular	0.3, 0.5, 0.7
		high	right-shoulder	0.6, 0.8, 1

**Table 4:** Example of fuzzy rules for the second FLS.

Rule Index	σ	υ	κ	τ	Decision
R1	medium	high	normal	high	cloud
R2	high	medium	heavy	low	cloud
R3	high	high	heavy	high	cloud
R4	low	low	light	high	remote edge
R5	low	medium	light	high	remote edge

**Table 5:** Simulation parameters [65].

Parameter	Value
Simulation time/warm-up period	33/3 min
Minimum/maximum number of IoT devices	250/2500
Step size of IoT device count	250
Number of edge/cloud servers	14/1
Number of VMs per edge/cloud server	8/4
Number of cores per edge/cloud VM CPU	2/4
VM CPU speed per edge/cloud	10/100 GIPS
Mobility model	Random way point
MAN bandwidth	MMPP/M/1 model
WAN/WLAN bandwidth	Empirical
LAN propagation delay	5 ms
Learning rate	0.001
Epsilon	0.1
Discount factor	0.5

**Table 6:** Parameters of application types [65].

	Healthcare	AR	Infotainment	Compute-Intensive
Usage percentage (%)	20	30	30	20
Task interval (sec)	3	2	7	4
Delay sensitivity	0.6	0.9	0.4	0.15
Active/Idle period (sec)	45/90	40/20	30/45	60/120
Upload/Download data (KB)	20/1250	1500/25	25/1000	2500/200
Task length (GI)	6	9	15	30
VM utilization on edge (%)	4	6	10	20
VM utilization on cloud (%)	0.4	0.6	1	2

## Data Availability

Not applicable.

## Abbreviations

The following abbreviations are used in this manuscript:

5G	Fifth generation
AR	Augmented reality
BWM	Best-worst method
COG	Center of gravity
CPU	Central unit processing
DNN	Deep neural network
ETSI	European Telecommunications Standards Institute
FCD	Fuzzy clustering defuzzification
FLS	Fuzzy logic system
GWO	Grey wolf optimizer
IoT	Internet of Things
LAN	Local area network
LSTM	Long short-term memory
MAN	Metropolitan area network
MCC	Mobile cloud computing
MDP	Markov decision process
MEC	Multi-access edge computing
MEO	Mobile edge orchestrator
ML	Machine learning
MOM	Mean of maximum
PSO	Particle swarm optimization
QoE	Quality of experience
QoS	Quality of Services
SARSA	State-action-reward-state-action
TOPSIS	Technique for Order of Preference by Similarity to Ideal Solution
VEC	Vehicular edge computing
VM	Virtual machine
WAN	Wide area network
WFM	Weighted fuzzy mean
WLAN	Wireless local area network
